# The Validity and Reliability of the Persian Version of the Xerostomia Inventory Questionnaire

**DOI:** 10.30476/dentjods.2023.95143.1839

**Published:** 2024-03-01

**Authors:** Maedeh Barkhordari Dashtkhaki, Shahla Kakoei, Mohammad Hasan Larizadeh, Amir Hossein Nekouei

**Affiliations:** 1 Oral and Dental Diseases Research Center, Kerman University of Medical Sciences, Kerman, Iran; 2 Dept. Oral and Dental Diseases Research Center, Kerman University of Medical Sciences, Kerman, Iran; 3 Dept. of Radiation Oncology, Neuroscience Research Center, Kerman University of Medical Sciences, Kerman, Iran; 4 Social Determinants on Oral Health Research Center, School of Dentistry, Kerman University of Medical Sciences, Kerman, Iran

**Keywords:** Xerostomia Inventory questionnaire, Validity, Reliability, Translation

## Abstract

**Statement of the Problem:**

Reduced saliva production or changes in the quality of saliva are major causes of xerostomia as a perceptual disease.

**Purpose:**

This study aimed to measure validity and reliability of the Persian version of the xerostomia inventory (XI) questionnaire.

**Materials and Method:**

This cross-sectional study was performed in Kerman in 2021. The sample size for this study was 100 people. To test the discriminant validity of XI, 50 healthy people (control group) and 50 people undergoing or recovering from laryngeal radiation (xerostomia group) were chosen. Cronbach's alpha coefficient was used to determine internal consistency, and the intra-cluster correlation (ICC) coefficient was used to determine test-retest reliability after two weeks. To evaluate the concurrent validity, the relationship between the total score of the questionnaire and the golden question, which was defined as “How often do you have dry mouth?” was measured.

**Results:**

Cronbach's alpha and ICC coefficient for the total XI score were 0.84 and 0.95, respectively. The mean ages of patients in the radiotherapy group and the healthy participants were 59±7.5 and 41.1±6.6 years, respectively. Participants who were having or had had laryngeal radiotherapy had a significantly higher mean total XI score than healthy individuals (*p*< 0.001).

**Conclusion:**

The Persian version of the Xerostomia Inventory is a valid and reliable tool for assessing xerostomia.

## Introduction

Dry mouth (xerostomia) is a common disorder and a perceptual condition that affect the quality of life [ 1 - 2 ]. It is often caused by reduced saliva production or changes in the quality of saliva [ 1 , 3 ]. Xerostomia increases with age [ 4 ]. It is more prevalent in women and can be due to certain health conditions such as infection, inflammation, malignancy in the salivary glands, autoimmune diseases, mental health problems (depression), diabetes, infectious diseases (hepatitis and AIDS), consumption of some medications (such as antihypertensives, antidepressants, sedatives, anxiolytics), radiation therapy, and chemotherapy [ 5 ]. Also, xerostomia can occur as a result of drug and alcohol use [ 3 , 6 ]. Moreover, xerostomia can cause conditions such as papillary atrophy of the tongue, cavities, periodontal diseases, lesions, mucosal pallor, dry lips, angular cheilitis, fungal infections such as candidiasis, and taste disorders [ 7 - 10 ]. 

Xerostomia is diagnosed using the patient's history, clinical examination, spitting test, and sialometry. The most common method for collecting saliva is the spitting test [ 11 ]. The average normal stimulated and unstimulated salivary flow rates are 1.5–2 ml/min, and 0.3–04 ml/min, respectively [ 12 ]. Most researchers consider unstimulated saliva flow < 0.1 ml/min and stimulated saliva flow < 0.7 ml/min to be abnormal and a sign of hyposalivation [ 13 ]. Questionnaires can indicate the patient's own experience of the condition [ 14 ]. Several xerostomia questionnaires, such as the Summarized Xerostomia Inventory (SXI) and Xerostomia Questionnaire (XQ), have been developed [ 14 - 17 ]. 

The Xerostomia Inventory (XI) tool was validated and translated to Turkish by Keskin *et al*. [ 18 ], German by Hohenberger *et al*. [ 19 ], Portuguese by Amaral *et al*. [ 20 ], and also by Da Mata *et al*. [ 21 ], Korean by Lee *et al*. [ 22 ], Spanish by Serrano *et al*. [ 23 ], and Greek by Gkavela *et al*. [ 24 ]. Moreover, Jiang *et al*. [ 25 ] and Lin *et al*. [ 26 ] validated the Chinese and Taiwanese versions of the XQ, respectively. Furthermore, the Indonesian and Dutch versions of the SXI were validated by Wimardhani *et al*. [ 27 ] and Thomson *et al*. [ 28 ], respectively. The diagnosis of xerostomia is essential because it is a common disorder affecting patients' quality of life [ 5 , 29 ]. The spitting test to diagnose this condition has been limited due to COVID-19.

According to our research, the XI has not been translated and validated in Iran. Effective management of xerostomia is possible with a valid and sensitive measurement tool; therefore, this study was conducted to translate the XI questionnaire to Persian and validate the translation.

## Material and Method

This cross-sectional study was conducted in Kerman in 2021. This study aimed to assess the validity and reliability of the Persian version of the XI. The Kerman Medical University Ethics Committee approved this study with IR's ethics code KMU.REC.1400.287. All personal information, such as their contact number, first name, and surname, was removed from all files, and the files were provided to the statistical consultant for analysis after blinding. Moreover, participating in the project was voluntary, and verbal informed consent was obtained from the participants. 

### The translation of the questionnaire

First, the back-translation method was used to translate the questionnaire to Persian [ 30 - 31 ]. The original English version of the questionnaire was first translated to Persian by two people proficient in specialized English. Then, the translated version was re-translated to English by two bilingual translators who were proficient in translating Persian to English and had never seen the original version of the questionnaire. These three versions (the original English version, Persian translation, and English translation) were reviewed by three dentistry experts. The contradictions and inconsistencies between the translations were eliminated and lastly, the final questionnaire was prepared. The questionnaires were anonymous, and participants were assured that their personal information would only be used for statistical analysis.

### Participants

The study participants were divided into two groups including those who were having or had radiotherapy with laryngeal cancers and those who had not received any form of radiotherapy. The contact numbers of 95 laryngeal cancer patients who were having or had radiotherapy in the radiotherapy center of Shafa Hospital of Kerman were randomly selected, maintaining the confidentiality of information and details. After contacting them and checking the inclusion and exclusion criteria, first, the verbal informed consent text form was read over the telephone, and after obtaining the consent, 50 of them were recruited. Similarly, the contact numbers of 120 patients referred to the dentistry school for dental treatments were randomly selected. Afterward, 120 of those patients were contacted, the inclusion and exclusion criteria were checked, the verbal informed consent was obtained, and 70 of them were recruited.

### Inclusion-Exclusion criteria

All subjects between the ages of 20 and 70 were included in the study. Patients were included in the study if they had completed at least 25 head and neck radiotherapy sessions (they had completed radiotherapy or still were receiving radiotherapy). Healthy participants with a history of head and neck radiotherapy, diabetes, Sjögren’s syndrome, HIV, hepatitis C, graft versus host disease (GVHD), chemotherapy, autoimmune diseases, Bell’s palsy, taking antidepressants (such as Amitriptyline, Citalopram, Fluoxetine, Paroxetine, Sertraline, Bupropion, and so on), diuretic antihypertensive ( such as Reserpine, Methyldopa, Chlorothiazide, Furosemide, Metoprolol, calcium channel blockers, and so on), antihistamines (such as Diphenhydramine, Chlorphenamine and so on), antipsychotics (such as derivatives of Phenothiazine, Haloperidol, and so on), sedatives and anxiolytics (such as Diazepam, Lorazepam, and so on), as well as smokers and those with mouth breathing, were excluded from the study. 

### Participant recruitment and Questionnaire Piloting

First, the questionnaire was completed for 20 healthy individuals by telephone in a pilot study. These participants were asked to comment on any problems in understanding the questionnaire.

### Assessing the reliability and validity of the questionnaire

The final questionnaire was completed by 50 patients with laryngeal cancer who had or were having radiotherapy and 50 healthy clients of the dentistry school over the telephone. A family member interviewed patients who were unable to answer or speak due to laryngeal problems, and their answers were then repeated to the interviewer. The questionnaire was completed after two weeks in the group of healthy individuals over the telephone to assess the reliability. Cronbach's alpha was used to measure internal consistency and overall reliability. The lowest acceptable value for Cronbach's alpha was 0.7. Concurrent validity was measured simultaneously using the standard item for measuring xerostomia, "How often do you have dry mouth?" and the XI questionnaire score for all participants. The responses for this single item were "Never”, "Hardly ever”, "Occasionally", "Fairly often", and "Very often". The temporal reliability of the scale was measured using the intraclass correlation coefficient (ICC) and test-retest method by re-evaluating the group of healthy individuals after two weeks under similar conditions. 

### Statistical Analysis

Statistical analysis was conducted by analysis of covariance (ANCOVA), ICC, Spearman's correlation coefficient, and Cronbach's alpha using SPSS 23. The significance level in this study was considered 0.05%. 

## Results

In the present study, after the translation of the questionnaire to Persian, the word cough lollies were replaced with the word sour candy because Iranians are not familiar with the word cough lollies. No other problems were observed in other questions due to the simplicity of the English version. After the re-translation of the Persian version, no inconsistencies were detected between the original and re-translated versions. 

The mean age of the participants with xerostomia due to radiotherapy was 7.5±59, and the mean age of the healthy individuals was 6.6±41.1. Those who had undergone radiotherapy had an average age of 3.9±32.6 (Table 1). Moreover, 88% of the patients had completed their radiotherapy, and 12% were in the final stages of their radiotherapy. 

**Table 1:** The participants' demographic characteristics

		Sex				Age	
		Female		Male		Mean	Dispersion
		N	%	N	%		
Status	Individuals with xerostomia	3.0	6.0	47.0	94.0	59.0	7.5
	Healthy Individuals	38.0	76.0	12.0	24.0	41.1	6.6

Cronbach's alpha was 0.84, which indicates a high level of reliability. This index was also calculated if any of the items were removed, and it remained above 0.8 to the end. Therefore, not responding to any of the items did not cause a significant change in Cronbach's alpha value (Table 2). The total score of xerostomia with the ICC was 0.95 with a confidence interval of 95% (0.91, 0.97), which indicates the high reliability of the questionnaire. Moreover, The ICC for each item except the fifth and seventh questions was higher than 0.70. Therefore, relying on the total score of the questionnaire will lead to a higher level of reliability.

**Table 2:** The overall Cronbach's alpha and Cronbach's alpha if an item is deleted

Item	Cronbach's alpha if an item is deleted
1. I sip liquids to aid in swallowing food	0.82
2. My mouth feels dry when eating a meal	0.82
3. I get up at night to drink	0.83
4. My mouth feels dry	0.81
5. I have difficulty eating dry food	0.81
6. I suck sweets or candy to relieve dry mouth	0.85
7. I have difficulty swallowing certain food	0.81
8. The skin of my face feels dry	0.85
9. My eyes feel dry	0.85
10. My lips feel dry	0.84
11. The inside of my nose feels dry	0.84
Total	0.84

The relationship between the golden item "How often do you have a dry mouth" and other questions was measured, which showed a significant direct relationship between this item and the total score of the first, second, third, fourth, fifth, seventh, tenth and eleventh questions (*p*< 0.001). The correlation coefficient for the first, second, third, fourth, fifth, and seventh questions was higher than 0.6, indicating a strong correlation (Table 3). 

**Table 3:** The relationship between the participants' responses to the items and the item of "How often do you have dry mouth?"

	Correlation coefficient of items with the standard item	*p* value
1. I sip liquids to aid in swallowing food	0.695**	< 0.001
2. My mouth feels dry when eating a meal	0.610**	< 0.001
3. I get up at night to drink	0.545**	< 0.001
4. My mouth feels dry	0.992**	< 0.001
5. I have difficulty eating dry foods	0.709**	< 0.001
6. I suck sweets or candies to relieve dry mouth	0.190	0.051
7. I have difficulty swallowing certain food	0.697**	< 0.001
8. The skin of my face feels dry	0.080	0.452
9. My eyes feel dry	0.080	0.421
10. My lips feel dry	0.414**	< 0.001
11. The inside of my nose feels dry	0.217*	0.037
Total score	0.856	< 0.001

** . Correlation is significant at the 0.01 level (2-tailed).

* . Correlation is significant at the 0.05 level (2-tailed).

The analysis of covariance revealed that participants with xerostomia have a higher total score than healthy participants (*p*< 0.001). The effect size obtained from this comparison was 0.206 (Tables 4-5). 

**Table 4:** Comparison of the mean score of xerostomia in patients undergoing radiotherapy due to radiotherapy and healthy individuals

Variables	Parameter	Coefficient	Confidence Interval 95%		
			Lower bound	Upper bound	Effect size
	Intercept	11.1	1.9	20.3	
Status	Individuals with Xerostomia	12.6	7.6	17.6	0.206
	Healthy Individuals	Reference			
Sex	Female	2.1	-1.9	6.0	0.011
	Male	Reference			
Age		0.1	-0.1	0.3	0.016

**Table 5:** The intraclass correlation coefficient (ICC)

	ICC	95% CI	
1. I sip liquids to aid in swallowing food	0.87	0.78	0.92
2. My mouth feels dry when eating a meal	0.93	0.87	0.96
3. I get up at night to drink	0.81	0.68	0.88
4. My mouth feels dry	0.85	0.75	0.91
5. I have difficulty eating dry food	0.67	0.48	0.80
6. I suck sweets or cough lollies to relieve dry mouth	0.70	0.52	0.82
7. I have difficulty swallowing certain food	0.54	0.31	0.71
8. The skin of my face feels dry	0.95	0.91	0.97
9. My eyes feel dry	0.85	0.75	0.91
10. My lips feel dry	0.91	0.85	0.95
11. The inside of my nose feels dry	0.90	0.83	0.94
Total score	0.95	0.91	0.97

## Discussion

In the present study, all the questionnaire items were translated to Persian. After translating and retranslating the 11 items of this questionnaire, no difference was detected between the parallel translations. In the translation stage, we replaced the word cough lollies with the word sour candy. Cronbach's alpha value in this study was 0.84, and the ICC for the total score of the questionnaire was 0.95. Additionally, the correlation of the total score of the questionnaire with the standard item "How often do you have dry mouth?" was 0.85. The data showed that the total score of this questionnaire could reliably differentiate between people with xerostomia and healthy people.

Internal consistency of the questionnaire was assessed using Cronbach's alpha coefficient, which was 0.84. This coefficient's acceptable range of values was 0.7 to 0.95 [ 32 ]. Therefore, the Persian version has a good level of internal consistency. Moreover, it was shown that this value would still be higher than 0.8 if any of the questionnaire items were removed. It should be noted that this value was 0.90 in a similar study on the Portuguese version of this questionnaire, which confirms the results of the present study [ 21 ]. Furthermore, this coefficient was 0.92 and 0.6 in German [ 19 ] and Korean [ 22 ] versions, respectively. The reliability of the questionnaire using the test-retest method and calculating the ICC indicated that the total score could have similar results for individuals over time.

The value of the ICC was calculated to be 0.95 for the retest after two weeks, which is statistically significant and is between 0.91 and 0.97, considered excellent as they are above 0.9, with a 95% probability [ 33 ]. This value had been reported in previous studies to be 0.94 and 0.85 [ 19 , 21 ]. The intervals between the Portuguese and German test-retest assessments were two weeks and three months, respectively [ 19 , 21 ]. After two weeks for the Korean version, this value was 0.82 for the retest [ 22 ]. The ICC value for each item varied from 0.54 to 0.95. This value ranged from 0.48 to 0.81 for each item in the study in the Korean version [ 28 ]. Moreover, in the Portuguese version, this value ranged from 0.74 to 0.91 for each item [ 21 ].

The correlation between the total score and the single item "How often do you have dry mouth?" resulted in a strong, direct, and significant correlation of 0.85. This value was 0.48 in the study on the German version [ 19 ]. The SXI contains five items. This is probably the reason that the correlation of some of the items in the original version of XI in our study is, to some extent, weak [ 16 ].

The total score of the individuals with head and neck radiotherapy experience was higher than healthy individuals. The same result was obtained in the German version [ 19 ]. It is strong evidence for the discriminant validity of the XI questionnaire.

One of the limitations of our study was the impossibility of performing saliva collection tests due to the prevalence of COVID-19. For some participants who were having or had radiotherapy and were unable to speak, a family member answered the interviewer's questions. If the questionnaire is provided to the respondents, it can be more accurate. Additionally, the concurrent validity of this questionnaire and quality of life questionnaires is of great importance and can be investigated in future studies.

## Conclusion

The Persian version of XI has internal consistency due to a Cronbach's alpha of 0.84 and a very high level of reliability due to the ICC of 0.95. Moreover, it has a high level of concurrent validity with the golden item "How often do you have dry mouth?" and can reliably distinguish between people with xerostomia and healthy people.

## References

[1] Furness S, Worthington HV, Bryan G, Birchenough S, McMillan R ( 2011). Interventions for the management of dry mouth: topical therapies. Cochrane Database Syst Rev.

[2] Rayman S, Dincer E, Almas K ( 2010). Xerostomia. Diagnosis and management in dental practice. N Y State Dent J.

[3] Kakoei S, Nekouei AH, Kakooei S, Najafipour H ( 2021). The effect of demographic characteristics on the relationship between smoking and dry mouth in Iran: a cross-sectional, case-control study. Epidemiol Health.

[4] Gerdin EW, Einarson S, Jonsson M, Aronsson K, Johansson I ( 2005). Impact of dry mouth conditions on oral health-related quality of life in older people. Gerodontology.

[5] Millsop JW, Wang EA, Fazel N ( 2017). Etiology, evaluation, and management of xerostomia. Clin Dermatol.

[6] Nekouei AH, Kakooei S, Najafipour H, Kakoei S ( 2021). Oral Health Determinants among Opium Users in Kerman, Iran. Addict Health.

[7] Ouanounou A ( 2016). Xerostomia in the Geriatric Patient: Causes, Oral Manifestations, and Treatment. Compend Contin Educ Dent.

[8] Ursache M, Grădinaru I, Nechifor M, Cherciu-Ciubotaru B ( 2006). Implication ale xerostomiei în dishomeostazia orală [Implications of xerostomia in oral dishomeostasis]. Rev Med Chir Soc Med Nat Iasi.

[9] Yamamoto K, Kurihara M, Matsusue Y, Komatsu Y, Tsuyuki M, Fujimoto T, et al ( 2009). Atrophic change of tongue papilla in 44 patients with Sjögren syndrome. Oral Surg Oral Med Oral Pathol Oral Radiol Endod.

[10] Nekouei AH, Kakooei S, Najafipour H, Kakoei S ( 2021). Oral Health determinants among opium users in Kerman, Iran. Addict Health.

[11] Glick M ( 2021). Burket's oral medicine.

[12] Villa A, Connell CL, Abati S ( 2015). Diagnosis and management of xerostomia and hyposalivation. Ther Clin Risk Manag.

[13] Navazesh M, Kumar SK ( 2008). University of Southern California School of measuring salivary flow: challenges and opportunities. J Am Dent Assoc.

[14] Dirix P, Nuyts S, Van den Bogaert W ( 2006). Radiation-induced xerostomia in patients with head and neck cancer: a literature review. Cancer.

[15] Eisbruch A, Kim HM, Terrell JE, Marsh LH, Dawson LA, Ship JA ( 2001). Xerostomia and its predictors following parotid-sparing irradiation of head-and-neck cancer. Int J Radiat Oncol Biol Phys.

[16] Santiago PHR, Song Y, Hanna K, Nair R ( 2020). Degrees of xerostomia? A Rasch analysis of the Xerostomia Inventory. Community Dent Oral Epidemiol.

[17] Van der Putten GJ, Brand HS, Schols JM, de Baat C ( 2011). The diagnostic suitability of a xerostomia questionnaire and the association between xerostomia, hyposalivation and medication use in a group of nursing home residents. Clin Oral Investig.

[18] Başakcı Çalık B, Gür Kabul E, Keskin A, Bozcuk S, Şenol H, Çobankara V ( 2021). Translation and validation of a Turkish version of the Xerostomia Inventory XI in patients with primary Sjögren’s syndrome. Turk J Med Sci.

[19] Hohenberger R, Baumann I, Plinkert PK, Brinster R, Krisam J, Affolter A, et al ( 2019). Validating the Xerostomia Inventory in a radiation-induced xerostomia population in German language. Oral Dis.

[20] Amaral JPAR, Marques DNDS, Thomson WM, Vinagre ARR, da Mata ADSP ( 2018). Validity and reliability of a Portuguese version of the Summated Xerostomia Inventory-5. Gerodontology.

[21] Da Mata AD, da Silva Marques DN, Freitas FM, de Almeida Rato Amaral JP, Trindade RT, Barcelos FA, et al ( 2012). Translation, validation, and construct reliability of a Portuguese version of the Xerostomia Inventory. Oral Dis.

[22] Lee J, Koh JH, Kwok SK, Park SH ( 2016). Translation and validation of a Korean Version of the Xerostomia Inventory in Patients with Primary Sjögren's Syndrome. J Korean Med Sci.

[23] Serrano C, Fariña MP, Pérez C, Fernández M, Forman K, Carrasco M ( 2016). Translation and validation of a Spanish version of the xerostomia inventory. Gerodontology.

[24] Gkavela G, Kossioni A, Lyrakos G, Karkazis H, Volikas K ( 2015). Translation and preliminary validation of the Greek version of the Xerostomia Inventory in older people. European Geriatric Medicine.

[25] Jiang N, Wei S, Mårtensson J, Zhao Y, Årestedt K ( 2021). Assessment of Radiation-Induced Xerostomia: Validation of the Xerostomia Questionnaire in Chinese Patients With Head and Neck Cancer. Cancer Nurs.

[26] Lin SC, Jen YM, Chang YC, Lin CC ( 2008). Assessment of xerostomia and its impact on quality of life in head and neck cancer patients undergoing radiotherapy, and validation of the Taiwanese version of the xerostomia questionnaire. J Pain Symptom Manage.

[27] Wimardhani YS, Rahmayanti F, Maharani DA, Mayanti W, Thomson WM ( 2021). The validity and reliability of the Indonesian version of the Summated Xerostomia Inventory. Gerodontology.

[28] Thomson WM, van der Putten GJ, de Baat C, Ikebe K, Matsuda K, Enoki K, Hopcraft MS, Ling GY ( 2011). Shortening the xerostomia inventory. Oral Surg Oral Med Oral Pathol Oral Radiol Endod.

[29] Minicucci EM, Pires RB, Vieira RA, Miot HA, Sposto MR ( 2013). Assessing the impact of menopause on salivary flow and xerostomia. Aust Dent J.

[30] Vosoughi AR, Roustaei N, Mahdaviazad H ( 2018). American Orthopaedic Foot and Ankle Society ankle-hindfoot scale: A cross-cultural adaptation and validation study from Iran. Foot Ankle Surg.

[31] Mahdaviazad H, Roustaei N, Masoumpour MB, Razeghinejad MR ( 2018). Psychometric properties of the Glaucoma Quality of Life-15 questionnaire: Use of explanatory factor analysis. J Curr Ophthalmol.

[32] Tavakol M, Dennick R ( 2011). Making sense of Cronbach's alpha. Int J Med Educ.

[33] Koo TK, Li MY ( 2016). A guideline of selecting and reporting intraclass correlation coefficients for reliability research. J Chiropr Med.

